# Supplementary figures and images for: Built environment impact on the per capita cycling frequency of family——Based on two-level hierarchical linear model
Source: PLoS One. 2022 May 12;17(5):e0267891. doi: 10.1371/journal.pone.0267891 (PMC9098036; doi:10.1371/journal.pone.0267891)

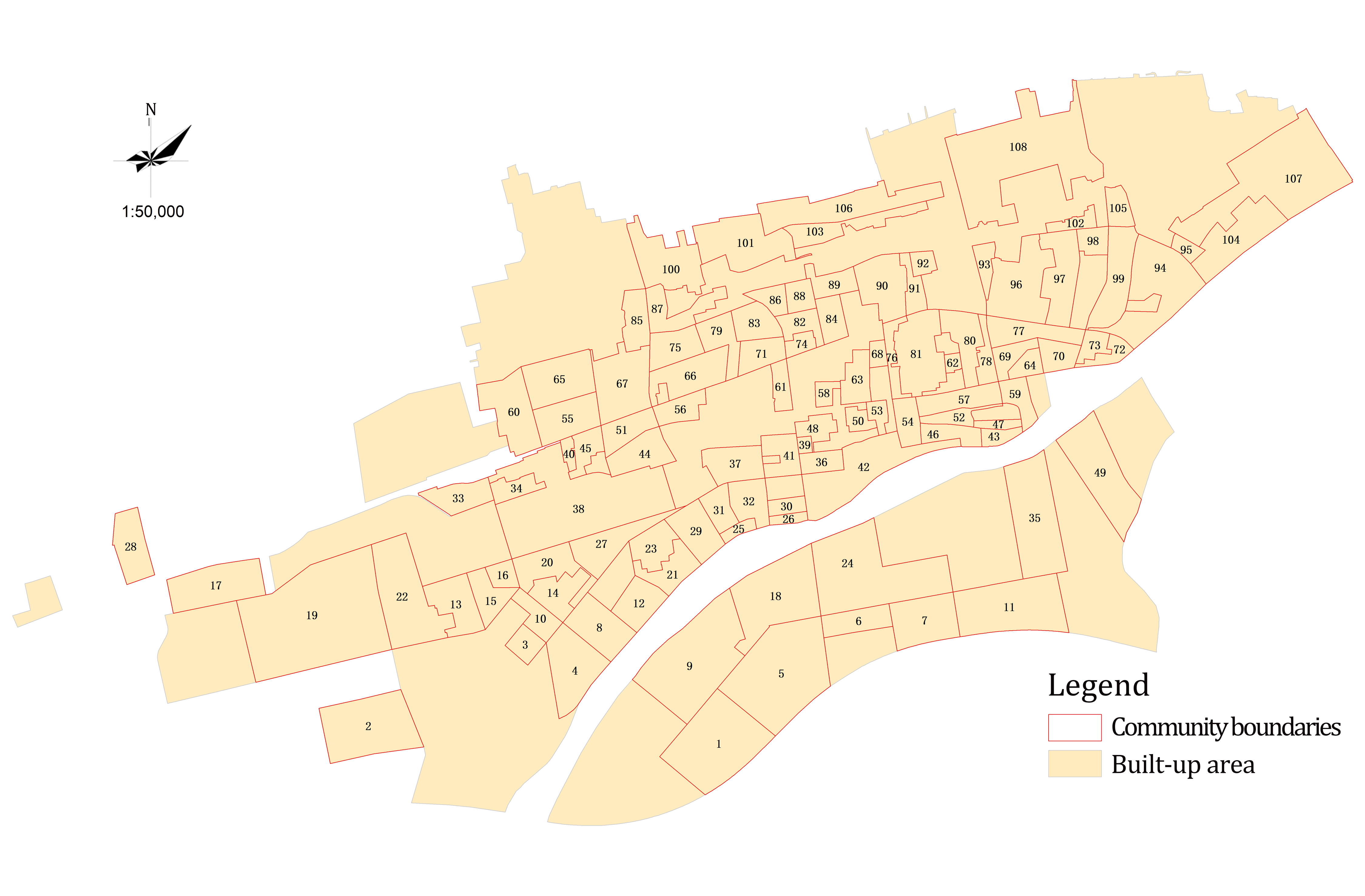

Supplement: S1 Fig — (TIF) [file pone.0267891.s001.tif]

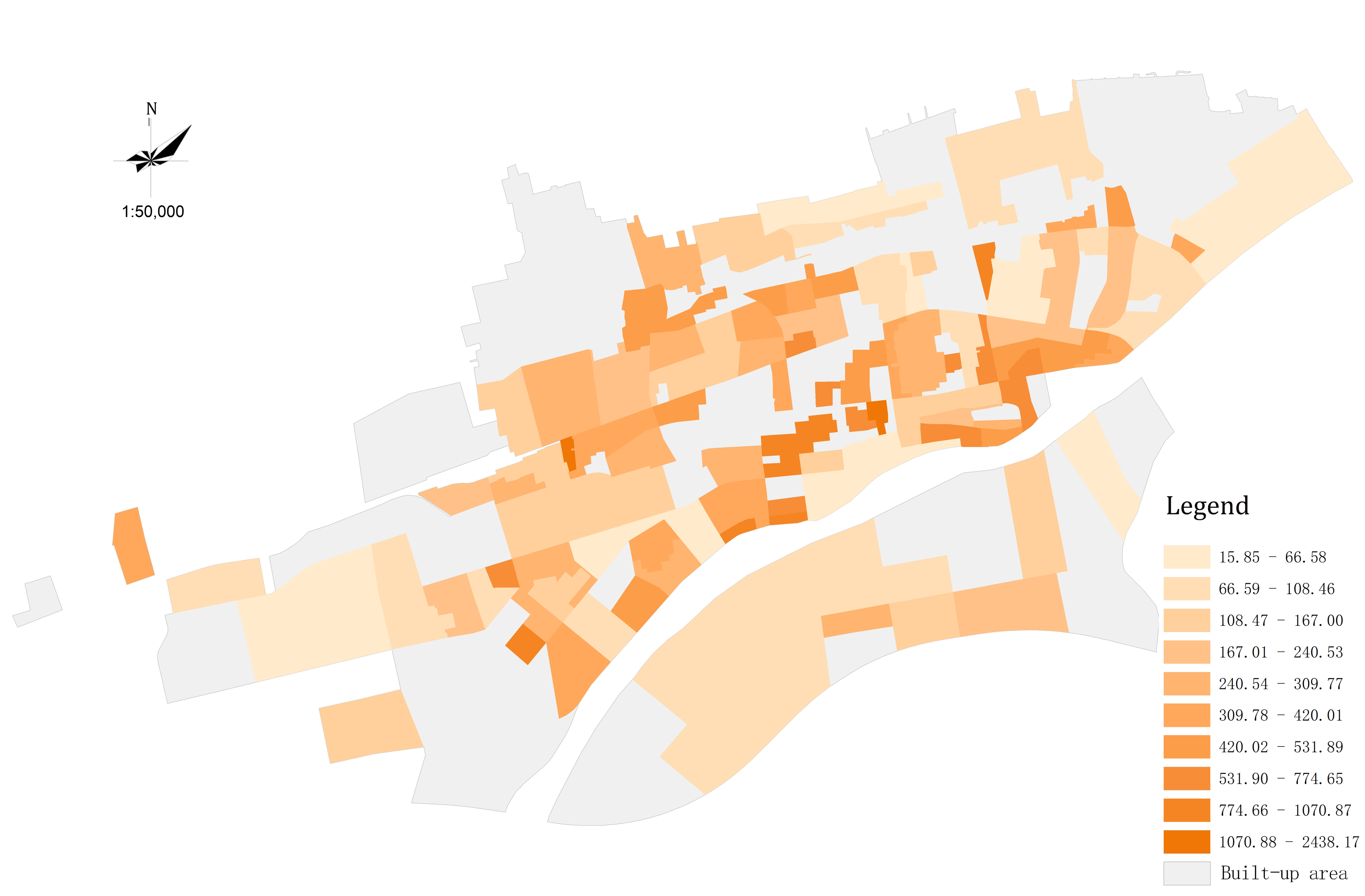

Supplement: S2 Fig — (TIF) [file pone.0267891.s002.tif]

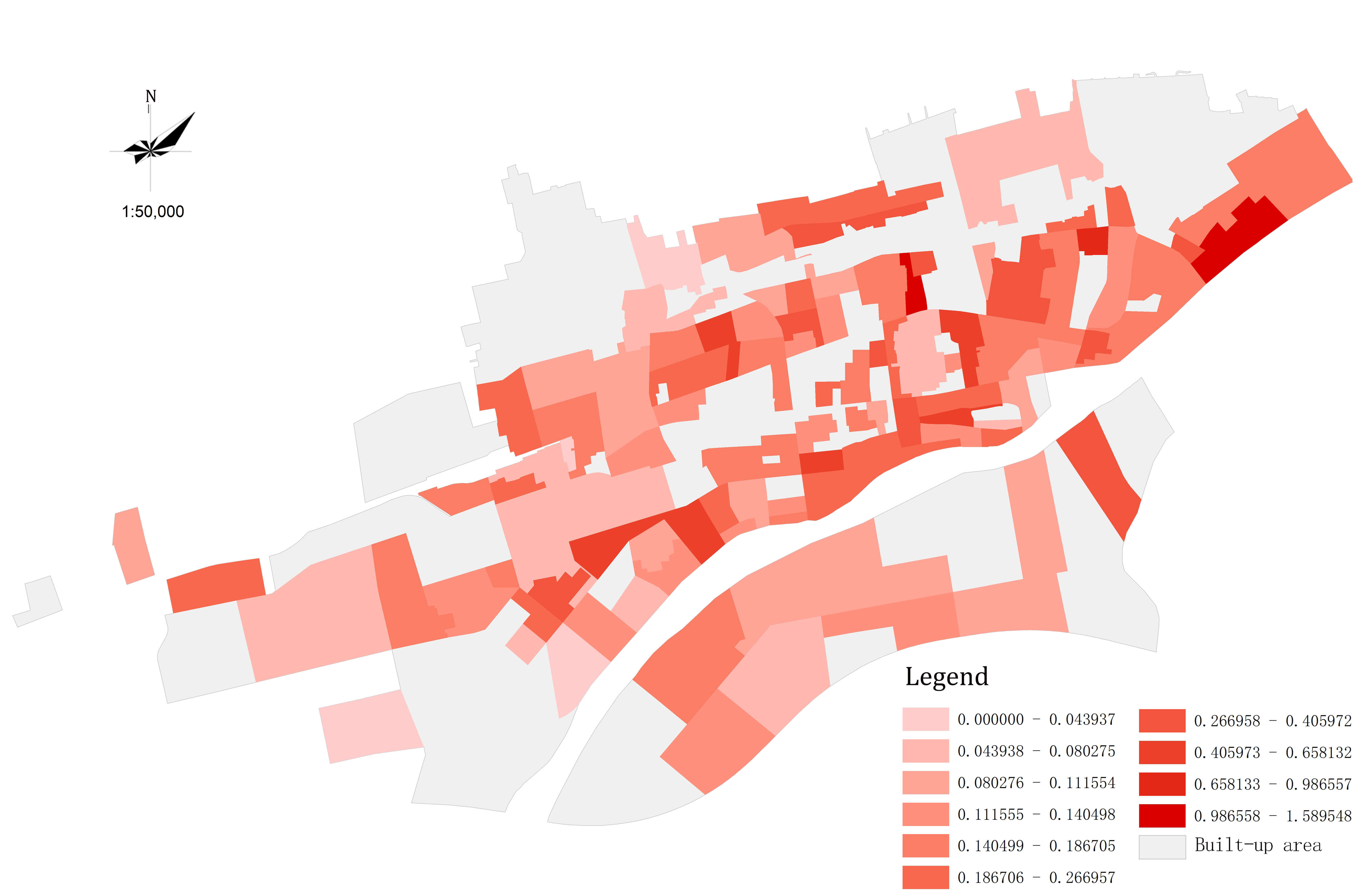

Supplement: S3 Fig — (TIF) [file pone.0267891.s003.tif]

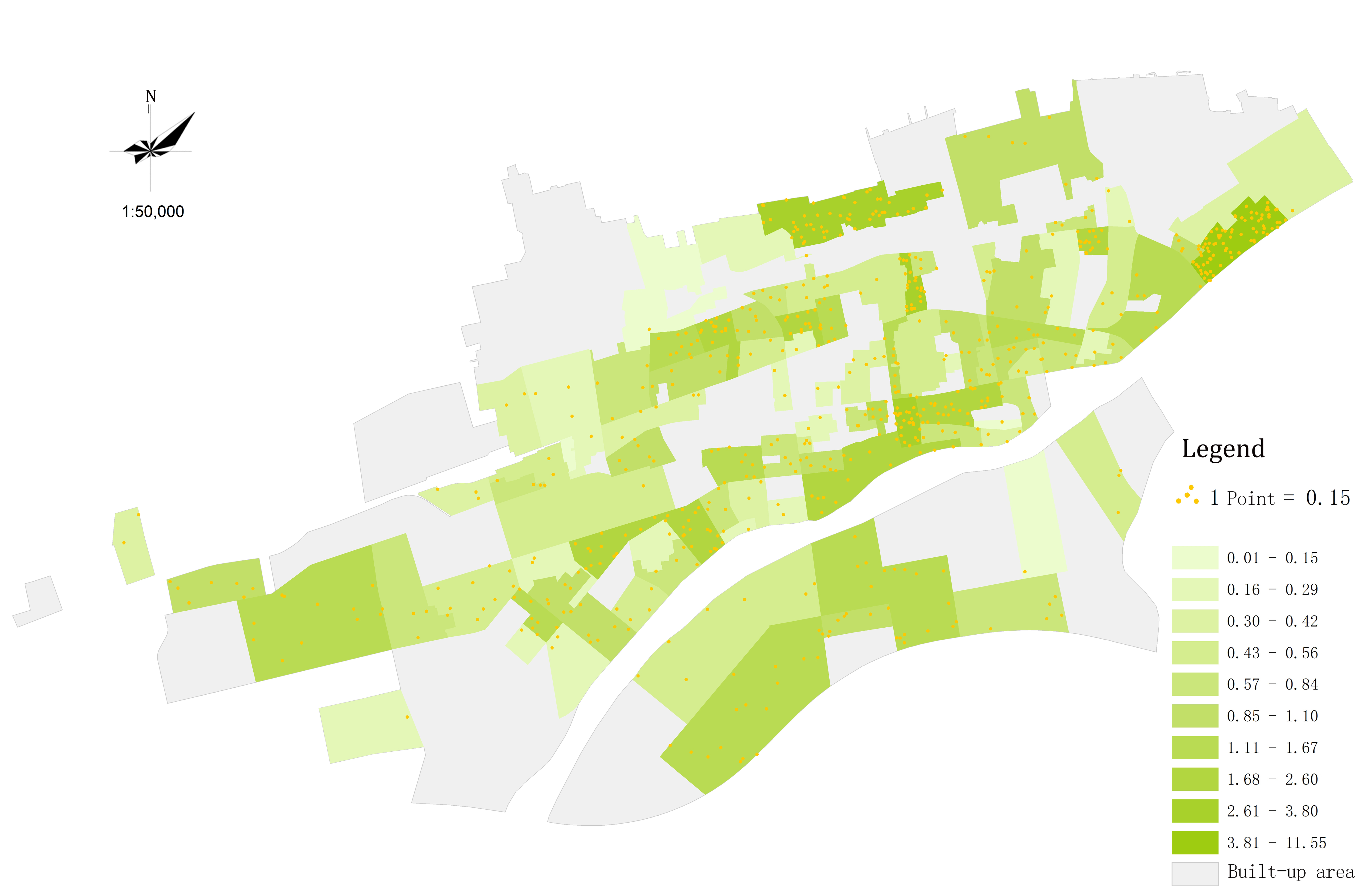

Supplement: S4 Fig — (TIF) [file pone.0267891.s004.tif]

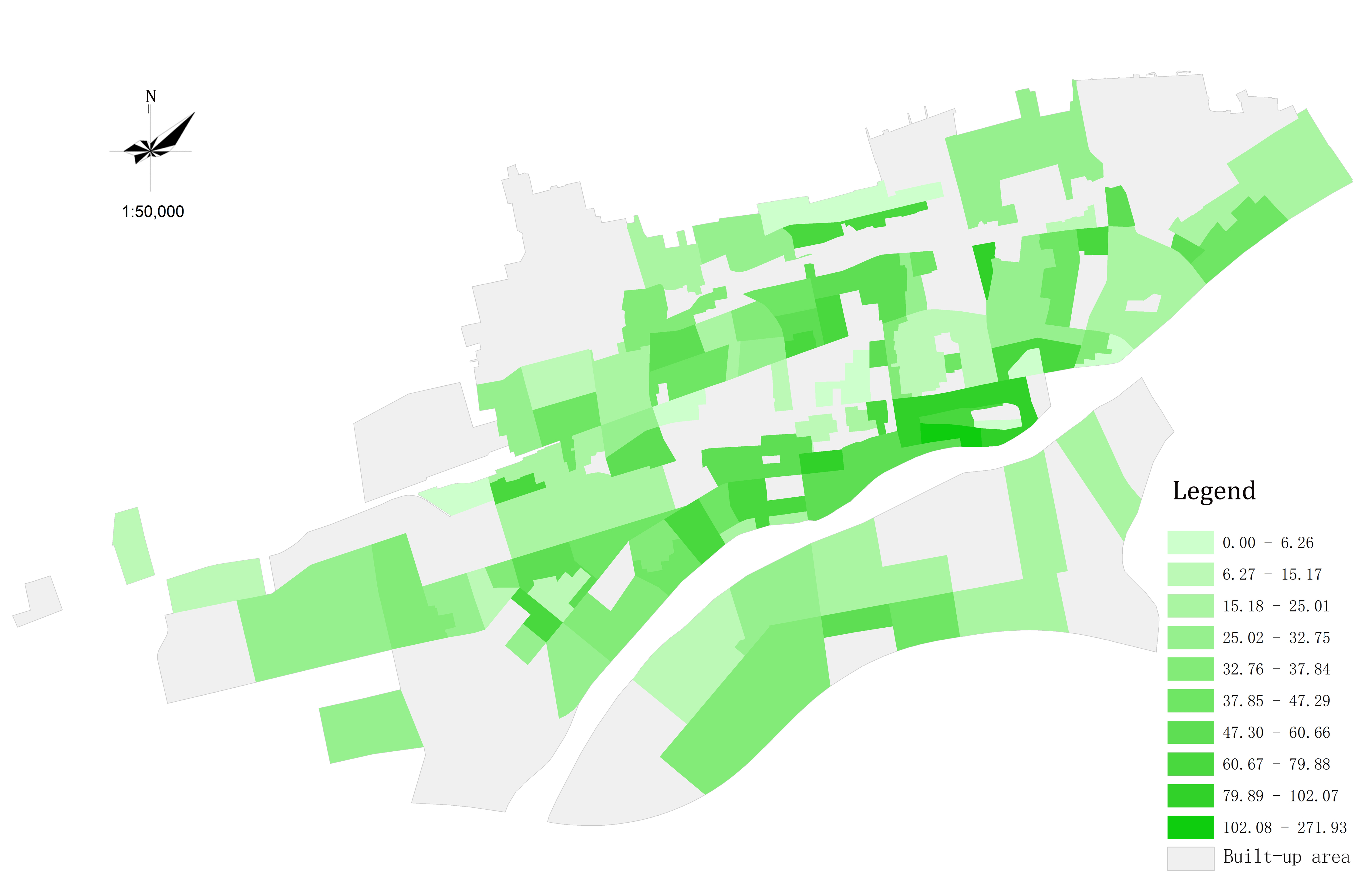

Supplement: S5 Fig — (TIF) [file pone.0267891.s005.tif]

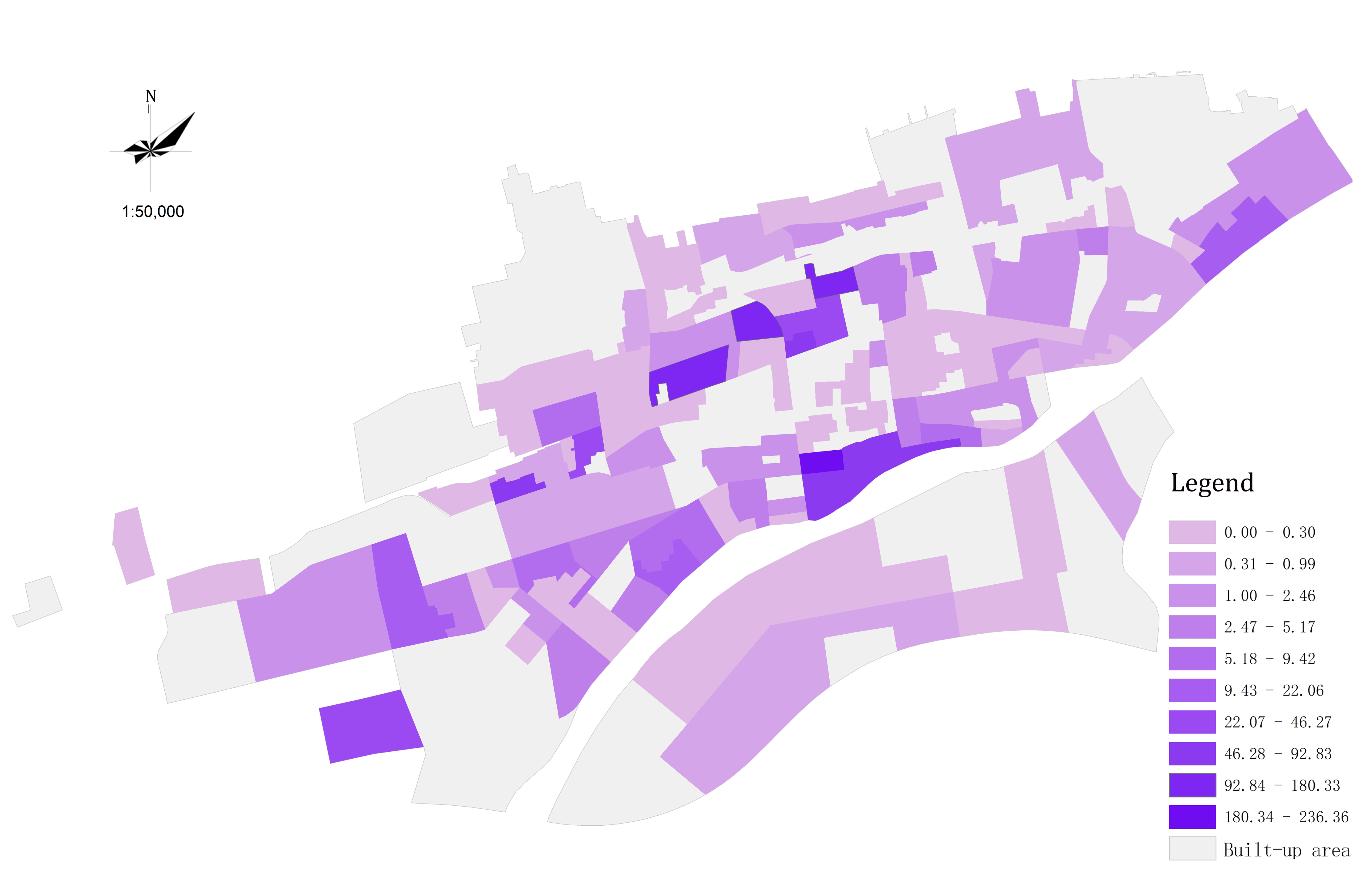

Supplement: S6 Fig — (TIF) [file pone.0267891.s006.tif]

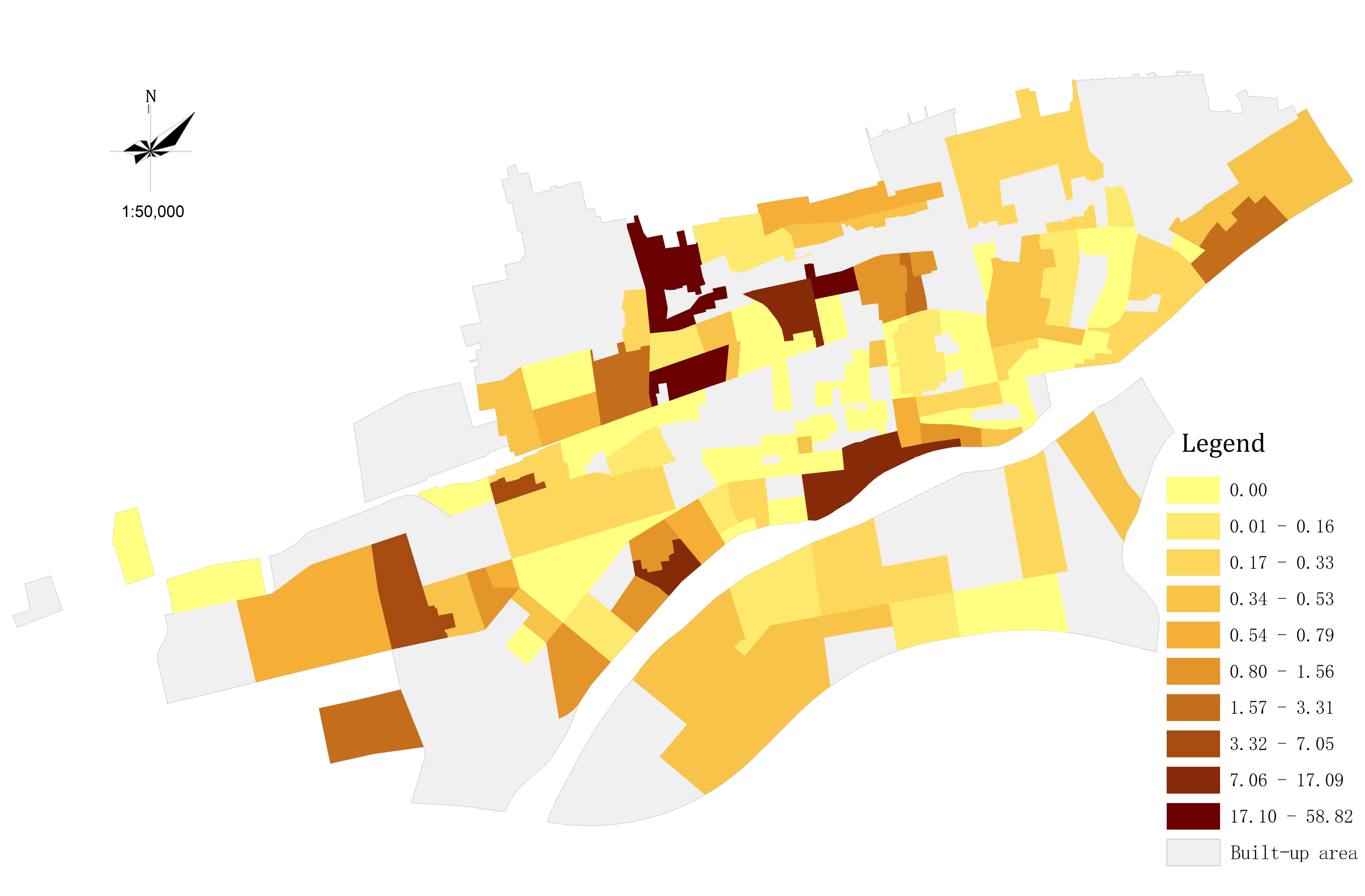

Supplement: S7 Fig — (TIF) [file pone.0267891.s007.tif]

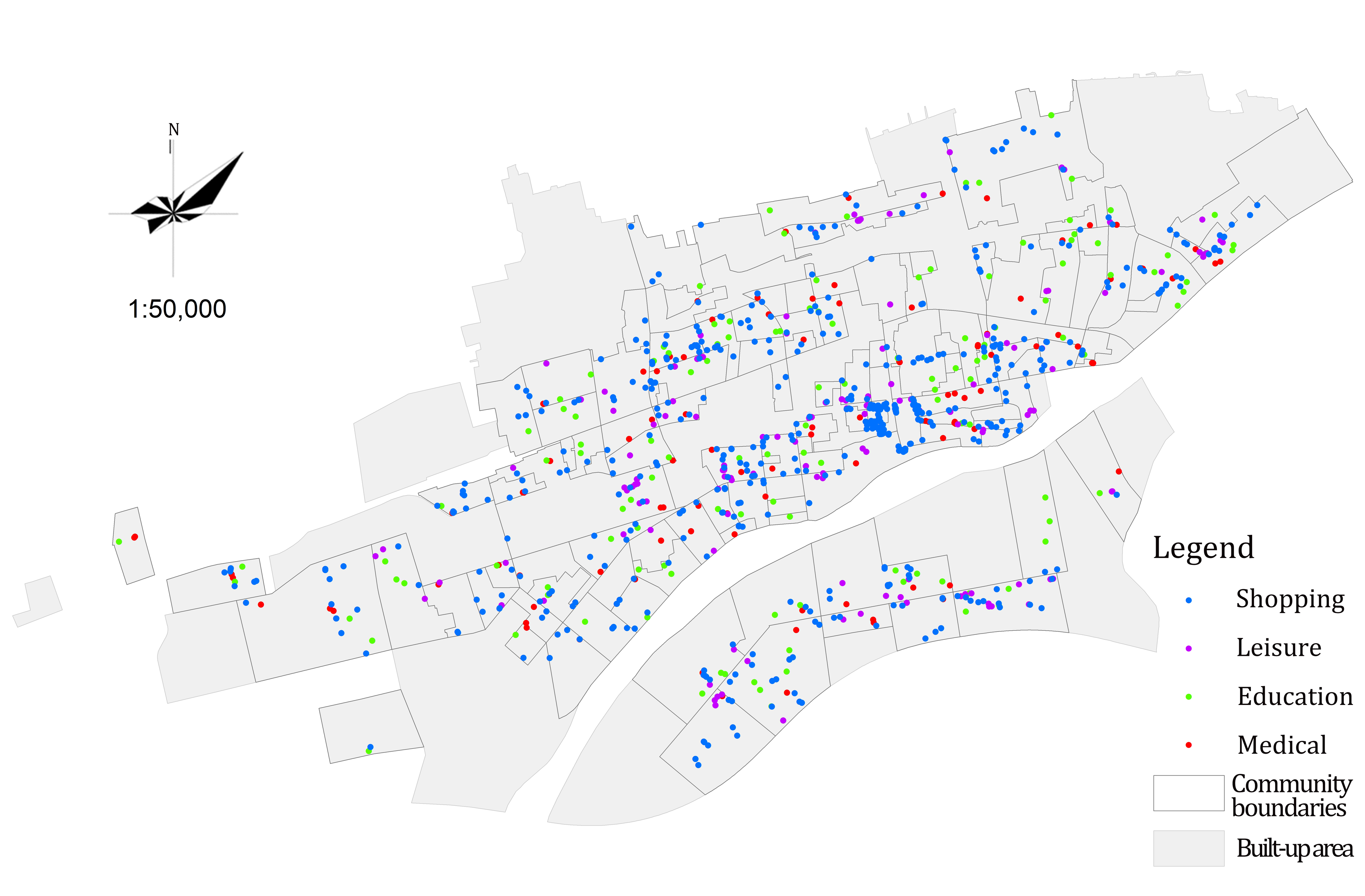

Supplement: S8 Fig — (TIF) [file pone.0267891.s008.tif]

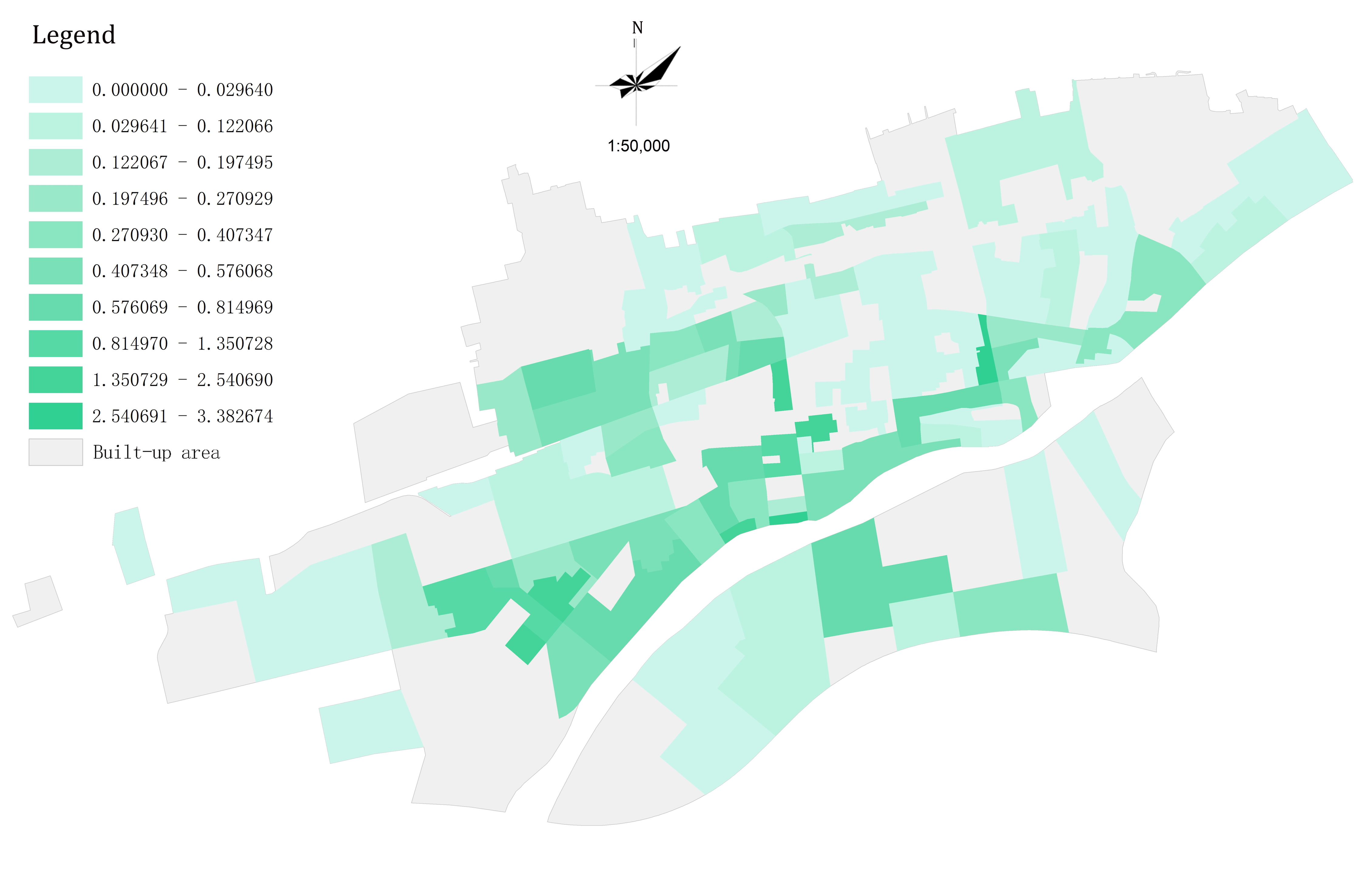

Supplement: S9 Fig — (TIF) [file pone.0267891.s009.tif]

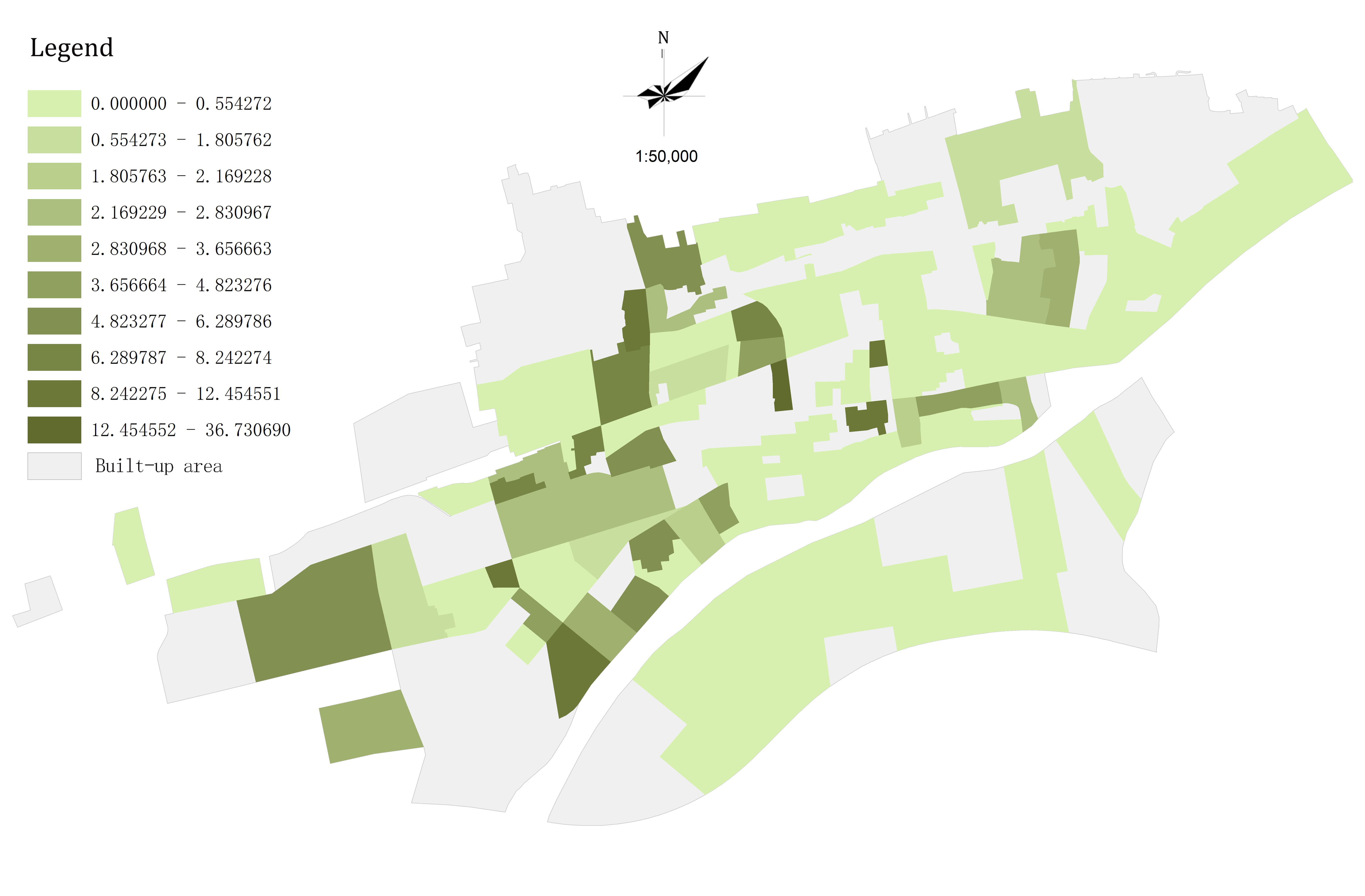

Supplement: S10 Fig — (TIF) [file pone.0267891.s010.tif]
